# MiR-34c suppresses tumor growth and metastasis in nasopharyngeal carcinoma by targeting MET

**DOI:** 10.1038/cddis.2014.582

**Published:** 2015-01-22

**Authors:** Y-Q Li, X-Y Ren, Q-M He, Y-F Xu, X-R Tang, Y Sun, M-S Zeng, T-B Kang, N Liu, J Ma

**Affiliations:** 1 Sun Yat-sen University Cancer Center, State Key Laboratory of Oncology in South China, Collaborative Innovation Center of Cancer Medicine, Guangzhou, Guangdong, China

## Abstract

Our previous microarray analysis indicated that miR-34c was downregulated in nasopharyngeal carcinoma (NPC). However, little is known about the function and molecular mechanism of miR-34c in NPC. In this study, miR-34c was found to be significantly downregulated in NPC cell lines and clinical tissues. Ectopic expression of miR-34c suppressed NPC cell viability, colony formation, anchorage-independent growth, cell migration and invasion *in vitro*, and inhibited xenograft tumor growth and lung metastasis *in vivo*. MET proto-oncogene (MET) was identified as a direct target of miR-34c using luciferase reporter assays, quantitative RT-PCR, western blotting and immunofluorescent staining. Overexpression of miR-34c markedly reduced MET expression at both the mRNA and protein levels. Knockdown of MET suppressed NPC cell proliferation, migration and invasion, whereas the restoration of MET rescued the suppressive effects of miR-34c. The demethylation agent 5-aza-2′-deoxycytidine (DAC) restored the expression of miR-34c in NPC cell lines. The promoter region of miR-34c was hypermethylated in NPC cells. In conclusion, miR-34c suppresses tumor growth and metastasis in NPC by targeting MET. The newly identified miR-34c/MET pathway provides further insights into the development and progression of NPC, and may represent a novel therapeutic target for NPC treatment.

Nasopharyngeal carcinoma (NPC) is an epithelial malignancy located in the nasopharynx. Globally, the highest incidence of NPC is observed in Southern China, where the rates vary from 20 to 50 cases per 100 000 people.^1, 2^ Although local and regional control have improved owing to the application of intensity-modulated radiotherapy and chemoradiotherapy, the prognosis for patients with NPC still remains very poor because of recurrence and distant metastasis.^3^ Therefore, understanding the molecular mechanisms that underlie the initiation and progression of NPC is of crucial significance to the development of novel therapeutic strategies.

MicroRNAs (miRNAs) are small noncoding RNAs that are 19–25 nucleotides long.^4^ These molecules negatively regulate gene expression by base pairing with the 3′-untranslated region (3′-UTR) of their target mRNA transcripts.^5, 6, 7^ It has been reported that miRNAs control a wide range of biological and pathological processes.^8, 9, 10, 11^ Additionally, miRNAs are dysregulated in most tumor types,^12, 13, 14^ and may function as either tumor suppressors or oncogenes.^15, 16, 17^ To date, several miRNAs have been identified to be dysregulated and affect cell growth, proliferation and metastasis in NPC, including miR-451, miR-29c, miR-26a and miR-9.^18, 19, 20, 21^ These findings indicate that miRNAs have important roles in nasopharyngeal tumorigenesis. Therefore, further knowledge of the functional effects and molecular mechanisms of miRNAs may help to elucidate the biological events regulating the development and progression of NPC.

Recently, our microarray analysis demonstrated that miR-34c was downregulated in NPC.^22^ However, the exact roles of miR-34c in NPC have not yet been elucidated. In this study, we investigated the biological functions and molecular mechanisms of miR-34c in NPC. We found that miR-34c was downregulated and could suppress tumor growth and metastasis in NPC. Additionally, MET proto-oncogene (MET) was identified as a functional target of miR-34c. Thus, the newly identified miR-34c/MET pathway expands our understanding of the mechanisms underlying the development and progression of NPC, and may provide a novel therapeutic target for the treatment of NPC.

## Results

### MiR-34c is downregulated in NPC cell lines and clinical specimens

**Figure 1:**
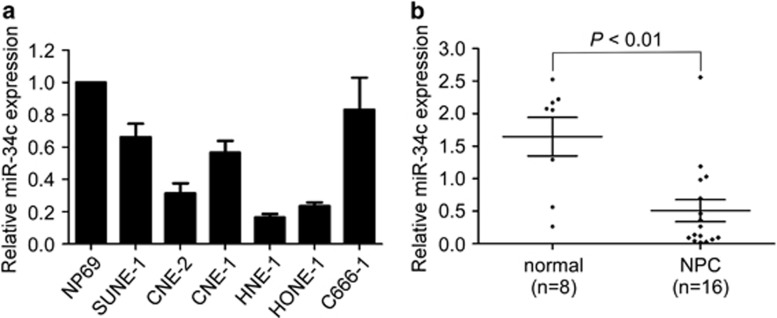
MiR-34c is downregulated in NPC cell lines and clinical specimens. (**a**) Relative expression of miR-34c in NP69 cells and NPC cell lines. Each experiment was independently repeated at least three times. (**b**) Relative expression of miR-34c in normal nasopharyngeal epithelial tissues (*n*=8) and NPC (*n*=16). U6 was used as an endogenous control. Data are presented as mean±S.D.; *P*-values were calculated using the Student's *t*-test

Recently, we observed that miR-34c was downregulated in NPC using microarray analysis.^22^ To confirm this result, we firstly quantified the expression of miR-34c in NPC cell lines and the normal nasopharyngeal epithelial cell line NP69, and found that miR-34c was significantly downregulated in NPC cell lines (Figure 1a). Furthermore, we evaluated the expression level of miR-34c in 16 freshly frozen NPC tissues and 8 normal nasopharyngeal epithelial tissues. MiR-34c expression was significantly reduced in NPC tissues (Figure 1b; *P*<0.01). These results suggest that miR-34c is downregulated in NPC.

### MiR-34c suppresses NPC cell proliferation and tumor growth *in vitro* and *in vivo*

**Figure 2:**
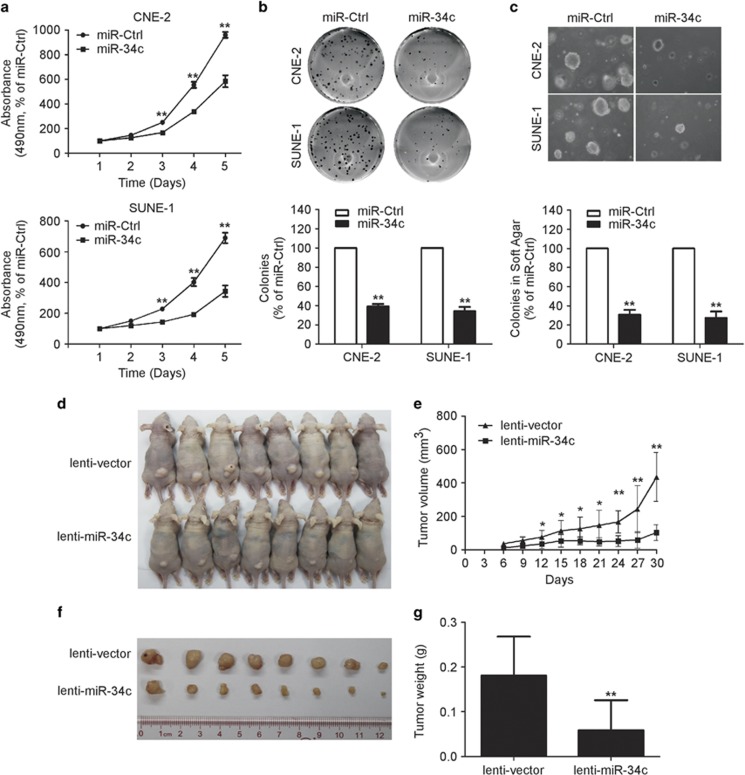
Restoring the expression of miR-34c suppresses NPC cell proliferation and tumor growth *in vitro* and *in vivo*. (**a**) MTT assay of CNE-2 and SUNE-1 cells transfected with miR-34c mimic (50 nM) or miR-Ctrl (50 nM). Each experiment was independently repeated at least three times. Data are presented as mean±S.D.; **P<0.01 compared with the miR-Ctrl group, Student's *t*-test (**b** and **c**) Representative results of the colony formation (**b**) and anchorage-independent growth (**c**) assays for CNE-2 or SUNE-1 cells transfected with miR-34c mimic or miR-Ctrl. Each experiment was independently repeated at least three times. Data are presented as mean±S.D.; **P<0.01 compared with the miR-Ctrl group, Student's *t*-test. (**d–g**) SUNE-1 cells stably overexpressing miR-34c (lenti-miR-34c) or negative control empty lenti-vector (lenti-vector) were subcutaneously injected into nude mice. (**d**) Four weeks later, the SUNE-1 cells stably overexpressing miR-34c had formed smaller tumors than the control cells. (**e**) Tumor volume growth curves. (**f**) Representative images of the excised tumors. (**g**) Tumor weight. Data are presented as mean±S.D.; *P<0.05 and **P<0.01 compared with the control lenti-vector group, Student's *t*-test

To determine whether ectopic expression of miR-34c affects the viability and proliferation of NPC cells, we performed the MTT assay and colony formation assay. CNE-2 and SUEN-1 cells transfected with miR-34c mimic displayed significant growth inhibition compared with cells transfected with miRNA negative control (miR-Ctrl) (Figure 2a; *P*<0.01). Additionally, the colony formation rate was remarkably lower in cells transfected with miR-34c mimic (Figure 2b; *P*<0.01).

Furthermore, we examined the effect of miR-34c on tumor growth using an *in vitro* soft-agar assay and an *in vivo* tumor growth model. *In vitro*, transfection with miR-34c mimic significantly inhibited the anchorage-independent growth ability of CNE-2 and SUNE-1 cells compared with those transfected with miR-Ctrl (Figure 2c; *P*<0.01). To investigate the effect of miR-34c on tumor growth *in vivo*, we created a xenograft tumor model by subcutaneously injecting SUNE-1 cells stably overexpressing miR-34c (lenti-miR-34c) or negative control empty lenti-vector (lenti-vector) into the dorsal flank of nude mice. Overexpression of miR-34c remarkably reduced tumor growth and tumor volume *in vivo* (Figures 2d and e; *P*<0.01), and the average tumor weight of the miR-34c-overexpressing group was also significantly lower than the control lenti-vector group (0.18±0.03 *versus* 0.06±0.23 g; Figures 4f and g; *P*<0.01). These results demonstrate that miR-34c suppresses NPC cell proliferation and tumor growth.

### MiR-34c suppresses NPC cell migration, invasion and metastasis *in vitro* and *in vivo*

**Figure 3:**
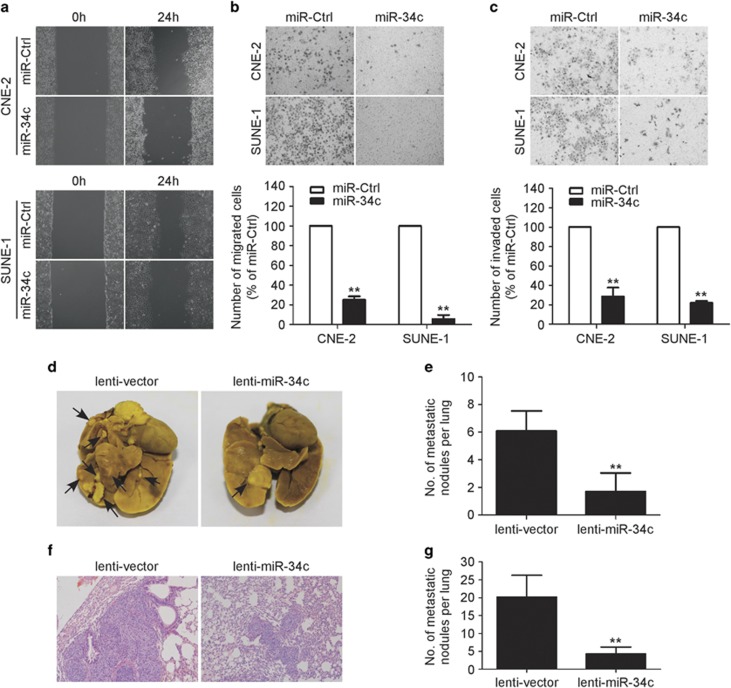
Restoration of miR-34c expression suppresses the migration, invasion and metastatic ability of NPC cells *in vitro* and *in vivo*. (**a–c**) Representative results of the wounding healing assay (**a**), Transwell migration assay (**b**) and Transwell invasion assay (**c**) for CNE-2 or SUNE-1 cells transfected with miR-34c mimic (50 nM) or miR-Ctrl (50 nM). Each experiment was independently repeated at least three times. Data are presented as mean±S.D.; **P<0.01 compared with the miR-Ctrl group, Student's *t*-test. (**d–g**) SUNE-1 cells stably overexpressing miR-34c (lenti-miR-34c) or negative control empty lenti-vector (lenti-vector) were intravenously injected via the tail vein and the formation of lung metastases was assessed after 8 weeks. Representative images (**d**) and quantification (**e**) of macroscopic metastatic nodules on the lung surface; arrowheads indicate metastatic nodules. Representative images (**f**) and quantification (**g**) of microscopic metastatic nodules in lung tissue sections stained with hematoxylin and eosin ( × 100). Data are presented as mean±S.D.; **P<0.01 compared with the control lenti-vector group, Student's *t*-test

To further explore whether ectopic expression of miR-34c affects the motility and invasion abilities of NPC cells *in vitro*, we performed wounding healing and Transwell assays. In the wound healing assay, cells transfected with miR-34c mimic migrated more slowly than those transfected with the miR-Ctrl (Figure 3a). In the Transwell assays, transfection of miR-34c mimic significantly reduced the migration and invasion abilities of CNE-2 and SUNE-1 cells (Figures 3b and c; *P*<0.01). These results suggest that ectopic expression of miR-34c suppresses NPC cell migration and invasion.

To further confirm the role of miR-34c in NPC cell invasion and metastasis *in vivo*, we conducted a lung metastasis assay by transplanting SUNE-1 cells stably overexpressing miR-34c or negative control empty lenti-vector via the tail vein. Eight weeks later, the lungs were dissected and the number of macroscopic and microscopic metastatic nodules was quantified. The number of macroscopic lung metastatic nodules was significantly lower in the miR-34c-overexpressing group than in the control lenti-vector group (Figures 3d and e; *P*<0.01). Similarly, H&E staining revealed that both the number and size of microscopic metastatic nodules were significantly lower in the miR-34c-overexpressing group (Figures 3f and g; *P*<0.01). Taken together, these findings suggest that miR-34c suppresses metastasis in NPC.

### MET is a direct target of miR-34c in NPC cells

**Figure 4:**
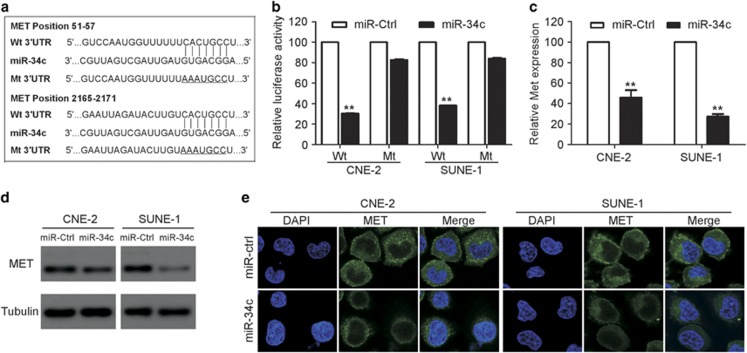
MET is a direct target of miR-34c in NPC. (**a**) Wt or Mt miR-34c target sequences of the MET mRNA 3′-UTR. (**b**) Relative luciferase activity of CNE-2 and SUNE-1 cells after co-transfection with Wt or Mt MET 3′-UTR reporter genes (2 *μ*g) and miR-34c mimic or miR-Ctrl (50 nM). Each experiment was independently repeated at least three times. Data are presented as mean±S.D.; **P<0.01 compared with the miR-Ctrl group, Student's *t*-test. (**c** and **e**) Quantification of MET mRNA expression by quantitative RT-PCR (**c**) and MET protein expression by western blotting (**d**) and immunofluorescent staining (**e**) after transfection with miR-34c mimic or miR-Ctrl. Each experiment was independently repeated at least three times. Data are presented as mean±S.D.; **P<0.01 compared with the miR-Ctrl group, Student's *t*-test

To further explore the molecular mechanism by which miR-34c exerts its biological function, we identified MET as a potential target of miR-34c using two publicly available databases (TargetScan and miRanda). To confirm whether MET is negatively regulated by miR-34c, we constructed luciferase reporter vectors containing the wild-type (Wt) or mutant (Mt) miR-34c target sequences of the MET 3′-UTR (Figure 4a). Overexpression of miR-34c significantly inhibited the luciferase activity of the Wt MET 3′-UTR reporter gene but not the Mt reporter gene (Figure 4b; *P*<0.01). In addition, overexpression of miR-34c markedly reduced the expression of MET at both the mRNA and protein levels (Figures 4c and e; *P*<0.01). These results demonstrate that MET is a direct target of miR-34c in NPC cells.

### MET is involved in miR-34c-regulated NPC cell proliferation, migration and invasion

**Figure 5:**
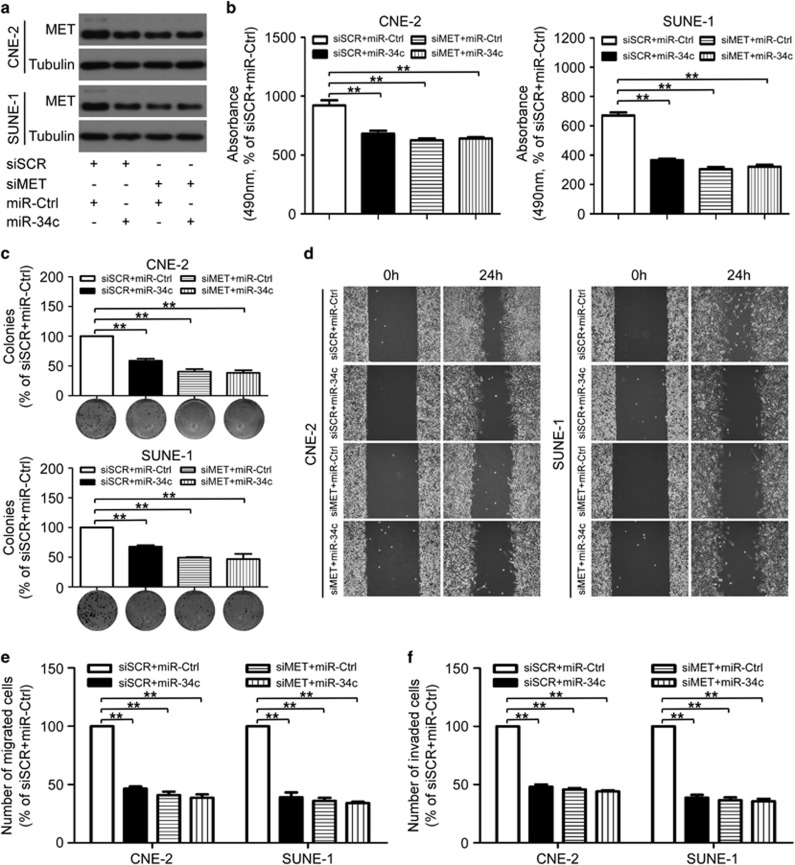
MiR-34c affects NPC cell proliferation, migration and invasion by directly targeting MET. (**a–f**) CNE-2 and SUNE-1 cells were co-transfected with siRNA (siMET or siSCR, 100 nM) and miRNA mimic (miR-34c mimic or miR-Ctrl, 50 nM). Each experiment was independently repeated at least three times. (**a**) Western blotting analysis of MET protein expression. (**b–e**) Knockdown of MET markedly inhibited the NPC cell proliferation, migration and invasion, and upregulation of miR-34c did not suppress proliferation, migration and invasion in siMET-transfected CNE-2 and SUNE-1 cells. Representative results of the MTT assay (**b**), colony formation assay (**c**), wounding healing assay (**d**), Transwell migration assay (**e**) and invasion assay (**f**). Data are presented as mean±S.D.; **P<0.01, Student's *t*-test

To determine the role of MET in the miR-34c-induced NPC cell growth and metastasis, CNE-2 or SUNE-1 cells were transiently co-transfected with siRNA (siMET or scrambled siRNA control (siSCR)) and miRNA mimic (miR-34c mimic or miR-Ctrl). Similar to the effects induced by overexpression of miR-34c, knockdown of MET significantly suppressed the cell viability, colony formation (Figures 5b and c; *P*<0.01), cell migration (Figures 5d and e; *P*<0.01) and invasion (Figure 5f; *P*<0.01), whereas overexpression of miR-34c did not have further suppressive effects on cell growth and metastasis in siMET-transfected CNE-2 and SUNE-1 cells (Figures 5b and f).

**Figure 6:**
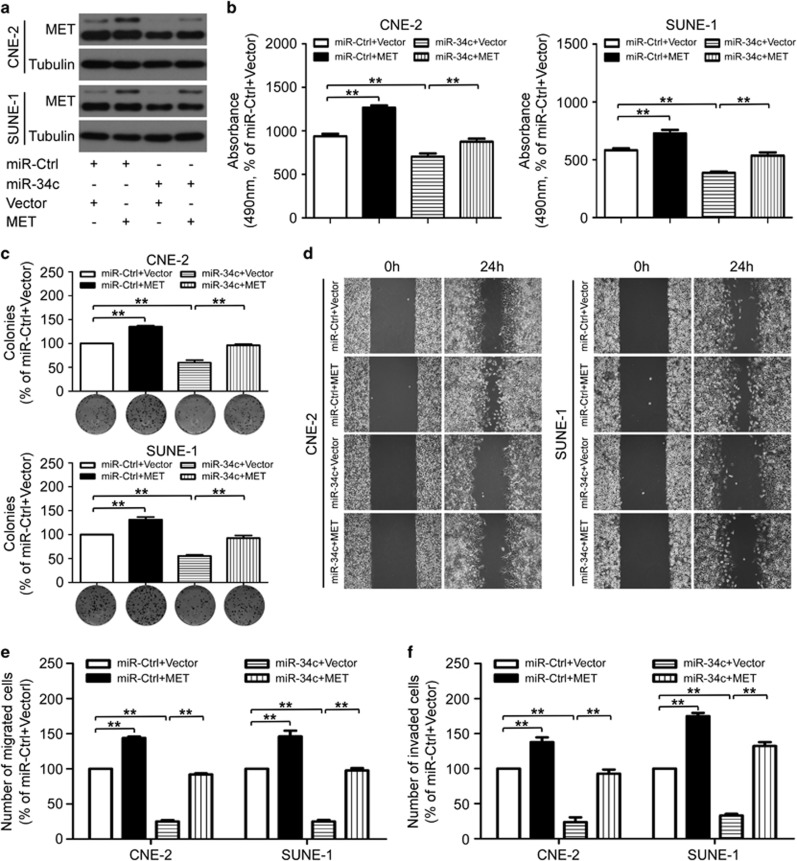
Overexpression of MET rescued the suppressive effects of miR-34c on NPC cell proliferation, migration and invasion. (**a–f**) CNE-2 and SUNE-1 cells were co-transfected with miRNA mimic (miR-34c mimic or miR-ctrl, 50 nM) and either the empty pReceiver-M02 vector control (Vector) or pReceiver-M02-MET plasmid- (MET) overexpressing MET (2 *μ*g). (**a**) Western blot analysis of MET protein expression. Each experiment was independently repeated at least three times. (**b–e**) Effects of restoration of MET on cell proliferation, migration and invasion. Representative results of the MTT assay (**b**), colony formation assay (**c**), wounding healing assay (**d**), Transwell migration assay (**e**) and invasion assay (**f**). Each experiment was independently repeated at least three times. Data are presented as mean±S.D.; **P<0.01, Student's *t*-test

To further confirm whether the tumor suppressive effects of miR-34c was directly mediated by MET in NPC, CNE-2 and SUNE-1 cells were co-transfected with miRNA mimic (miR-34c mimic or miR-ctrl) and either the empty pReceiver-M02 vector control (Vector) or pReceiver-M02-MET plasmid (MET), which encoded the full-length coding sequence of MET without its 3′-UTR (Figure 6a). Overexpression of MET abrogated the suppressive effects of miR-34c on proliferation (Figures 6b and c; *P*<0.01), migration (Figures 6d and e; *P*<0.01) and invasion (Figure 6f; *P*<0.01) in NPC cells. Taken together, these results demonstrate that MET is a direct and functional mediator for miR-34c in NPC.

### Promoter hypermethylation contributes to the downregulation of miR-34c in NPC

**Figure 7:**
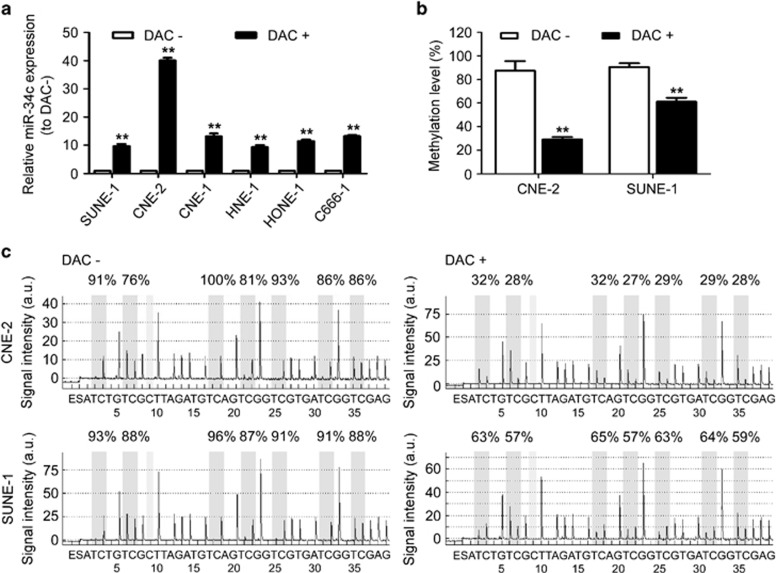
Downregulation of miR-34c in NPC cells is associated with promoter hypermethylation. (**a**) Relative expression of miR-34c in NPC cell lines treated with (+) or without (−) DAC (2 *μ*M). Each experiment was independently repeated at least three times. Data are presented as mean±S.D.; **P<0.01 compared with cells treated without (−) DAC, Student's *t*-test. (**b**) Average methylation level for seven CpG sites and (**c**) bisulfite pyrosequencing analysis of the miR-34c promoter region in CNE-2 and SUNE-1 cells treated with (+) or without (−) DAC. Data are presented as mean±S.D.; **P<0.01 compared with cells treated without (−) DAC, Student's *t*-test

To explore whether downregulation of miR-34c is associated with methylation aberrations, we firstly examined the expression of miR-34c in NPC cells treated with or without the demethylation agent DAC. We established that DAC treatment restored the expression of miR-34c in NPC cells (Figure 7a; *P*<0.01). To further investigate whether alerted miR-34c expression was due to methylation, we performed bisulfite pyrosequencing analysis. The promoter region of miR-34c was hypermethylated in CNE-2 and SUNE-1 cells, whereas the level of promoter methylation was significantly lower in cells treated with DAC (Figures 7b and c; *P*<0.01). These results indicate that downregulation of miR-34c in NPC cells may be associated with hypermethylation of the promoter region of miR-34c.

## Discussion

In the present study, we found that miR-34c was downregulated in NPC cell lines and clinical tissues. Ectopic expression of miR-34c suppressed NPC cell growth, migration and invasion *in vitro*, and inhibited tumor growth and metastasis *in vivo*. Furthermore, MET was identified as a direct target and functional mediator of miR-34c. Moreover, we provided evidence that the downregulation of miR-34c in NPC might be associated with hypermethylation of the promoter region of miR-34c. Taken together, these results suggest that downregulation of miR-34c has an important role in the development and progression of NPC.

MiRNAs are involved in the regulation of numerous cellular processes, and are frequently dysregulated in almost all types of cancers.^11, 12, 17^ Accumulating evidence indicates that a wide range of dysregulated miRNAs function as tumor suppressors or oncogenes, and contribute to the initiation and progression of cancer.^8, 15, 16^ To date, several studies have identified distinct miRNA expression profiles in NPC,^23, 24, 25^ and the dysregulation of specific miRNAs has been implicated in NPC development and progression.^18, 19, 20, 21^ Our recent microarray analysis indicated that the expression of miR-34c was significantly downregulated in NPC.^22^ However, the functions and mechanisms of miR-34c in NPC have not yet been elucidated. Thus, we conducted further investigations of miR-34c in NPC. First, quantitative RT-PCR demonstrated that the expression of miR-34c was significantly reduced in NPC cell lines and freshly frozen NPC tissue samples. These findings indicate that miR-34c is downregulated in NPC.

We then explored the biological function of miR-34c in NPC. Ectopic expression of miR-34c significantly suppressed the viability, and proliferative, migratory and invasive capabilities of NPC cells *in vitro*, and inhibited xenograft tumor growth and the formation of lung metastases *in vivo*. These results are consistent with observations in melanoma,^26^ lung,^27^ prostate^28^ and breast cancer.^29^ Recently, miR-34c has also been reported to be downregulated in several tumor types.^26, 27, 28, 29, 30^ Moreover, dysregulation of miR-34c has been proven to regulate tumor cell proliferation, apoptosis,^31^ senescence,^32^ migration and invasion,^26, 29^ which suggests that miR-34c exerts pivotal biological and pathological functions. The present study demonstrates that miR-34c suppresses NPC cell growth, migration and invasion, indicating that miR-34c may contribute to the development and progression of NPC.

MiRNAs exert their function by interacting with their target genes via base pairing to the 3′-UTR of mRNAs.^5, 6, 13^ Several genes have been identified as downstream targets of miR-34c, such as FRA-1 (Fos-related antigen 1),^29^ NOTCH4^33^ and MYC.^34^ In this study, we identified that MET was a direct target of miR-34c in NPC, in agreement with previous findings in prostate cancer.^29^ In addition, restoring the expression of miR-34c significantly reduced the expression of MET at both the mRNA and protein levels, and knockdown of MET phenocopied the suppressive effects of miR-34c on cell growth and metastasis, whereas ectopic expression of MET abrogated the suppressive effects of miR-34c in NPC cells. Recent studies have demonstrated that MET is overexpressed in a variety of cancer types, and this gene has been recognized as a key oncogene that promotes tumor growth, angiogenesis and metastasis.^35, 36, 37^ Several studies also have reported that overexpression of MET correlates with poor clinical outcome in patients with NPC.^38, 39^ Here, we found that silencing of MET significantly inhibited the viability, colony formation, migratory and invasive abilities of NPC cells. These results indicate that MET is a functional target of miR-34c, and that MET regulates NPC cell growth, migration and invasion.

Epigenetic aberrations, including DNA methylation and histone modification, are closely linked to altered miRNA expression.^40, 41^ Treatment with the demethylation agent DAC markedly restored the expression of miR-34c in NPC cells, indicating that aberrant DNA methylation may contribute to the downregulation of miR-34c in NPC cells. Recently, other studies showed that the promoter region of miR-34c was frequently hypermethylated, resulting in the downregulation of miR-34c in lung, gastric and colorectal cancer.^27, 30, 33, 42^ Similar to these findings, bisulfite pyrosequencing analysis demonstrated that the promoter region of miR-34c was hypermethylated in CNE-2 and SUNE-1 cells, and inversely, the expression of miR-34c was downregulated in these cells. Conversely, methylation of the miR-34c promoter was significantly reduced by DAC treatment in CNE-2 and SUNE-1 cells, leading to the upregulation of miR-34c. Taken together, these findings suggest that silencing of miR-34c in NPC cells may be due to hypermethylation of the promoter region of miR-34c.

In summary, our study demonstrates that miR-34c is downregulated in NPC, and miR-34c can suppress tumor growth and metastasis in NPC *in vitro* and *in vivo* by targeting MET. The newly identified miR-34c/MET pathway provides novel insight into the molecular mechanisms regulating progression and metastasis in NPC, and may provide novel therapeutic targets for the treatment of NPC.

## Materials and Methods

### Cell lines and clinical specimens

NP69 cells, a human immortalized nasopharyngeal epithelial cell line, were cultured in keratinocyte/serum-free medium (Invitrogen, Grand Island, NY, USA) supplemented with bovine pituitary extract (BD Biosciences, San Diego, CA, USA). Human NPC cell lines (CNE-1, CNE-2, C666-1, HNE-1, HONE-1 and SUNE-1) were maintained in RPMI-1640 (Invitrogen) supplemented with 10% FBS (Gibco, Grand Island, NY, USA); 293FT cells were grown in DMEM (Invitrogen) supplemented with 10% FBS.

Sixteen freshly frozen NPC samples and eight normal nasopharyngeal epithelium samples were collected from Sun Yat-sen University Cancer Center (Guangzhou, China). All samples were reviewed by pathologists to confirm the diagnosis. The research protocols were approved by the Institutional Ethical Review Board of Sun Yat-sen University Cancer Center, and informed consent was obtained from each patient.

### RNA extraction, reverse transcription and quantitative RT-PCR

Total RNA was extracted using TRIzol reagent (Invitrogen) as described previously,^23^ and reverse transcribed using M-MLV reverse transcriptase (Promega, Madison, WI, USA) with Bulge-Loop miRNA-specific RT primers (RiboBio, Guangzhou, China) for miR-34c or random primers (Promega) for MET. Quantitative RT-PCR reactions were performed in a CFX96 Touch sequence detection system (Bio-Rad, Hercules, CA, USA) using Platinum SYBR Green qPCR SuperMix-UDG reagents (Invitrogen). U6 or GAPDH were used as internal controls for miR-34c and MET, respectively, and the relative expression levels were calculated by the 2^−ΔΔCT^ method.^43^

### Oligonucleotide and plasmid transfection

CNE-2 and SUNE-1 cells were transfected with miR-34c mimic or miR-Ctrl (50 nM; GenePharma, Suzhou, China) using Lipofectamine 2000 reagent (Invitrogen). CNE-2 and SUNE-1 cells were transfected with siMET or siSCR (100 nM; GenePharma) using Lipofectamine 2000 reagent (Invitrogen). CNE-2 and SUNE-1 cells were co-transfected with the miR-34c mimic (50 nM) and either the pReceiver-M02-MET plasmid- (MET) overexpressing MET or empty pReceiver-M02 vector control (Vector) (2 *μ*g; FulenGen, Guangzhou, China) using Lipofectamine 2000 reagent (Invitrogen). The cells were harvested for assays 48 h after transfection.

### Generation of stably transfected cell lines

The pri-miR-34c sequence was cloned into the lentiviral plasmid pSin-EF2-puromycin (Addgene, Cambridge, MA, USA); pSin-EF2-miR-34c or negative control empty pSin-EF2 vector was then co-transfected into 293FT cells with the psPAX2 packaging plasmid (Addgene) and the pMD2.G envelope plasmid (Addgene) using the calcium phosphate method.^44^ At 24 h after transfection, lentiviruses expressing miR-34c (lenti-miR-34c) or negative control empty lenti-vector (lenti-vector) were harvested and used to infect SUNE-1 cells, and stably transfected cells were selected using puromycin and validated by quantitative RT-PCR.

### MTT, colony formation and anchorage-independent soft-agar assays

For the MTT assay, transfected CNE-2 or SUNE-1 cells were seeded at a density of 1000 cells per well in 96-well plates. At 1, 2, 3, 4 and 5 days, the absorbance values were measured at 490 nm using an ELX800 spectrophotometric plate reader (Bio-Tek, Winooski, VT, USA). For the colony formation assay, CNE-2 or SUNE-1 cells were plated at a density of 500 cells per well in six-well plates after transfection, and cultured for 7 or 12 days. The colonies were fixed in 4% paraformaldehyde, stained with 0.5% crystal violet and counted. For the soft-agar assay, 2.5 × 10^4^ transfected CNE-2 or SUNE-1 cells were suspended in 1 ml of complete medium containing 0.66% agar (Sigma-Aldrich, Ronkonkoma, NY, USA), and then placed on top of a layer of complete medium containing 1% agar in six-well plates; colonies were counted using an inverted microscope after 7 or 12 days.

### Wound healing, migration and invasion assays

For the wound healing assay, transfected CNE-2 or SUNE-1 cells were seeded into 6-well plates, subjected to serum starvation for 24 h in serum-free media, then an artificial wound was created in the confluent cell monolayer using a 200 *μ*l pipette tip and images were taken at 0 and 24 h using an inverted microscope. Migration and invasion assays were performed in Transwell chambers (Corning, Corning, NY, USA) coated without or with Matrigel (BD Biosciences) on the upper surface of the 8-*μ*m pore size membrane. Briefly, transfected CNE-2 or SUNE-1 cells were harvested, suspended in serum-free medium and 5 × 10^4^ or 1 × 10^5^ cells were plated into the upper chamber for the migration or invasion assays, respectively, and media supplemented with 10% FBS was placed into the lower chamber. After 12 or 24 h incubation, the cells that had migrated or invaded through the membrane to the lower surface were fixed, stained and counted using an inverted microscope.

### *In vivo* tumor growth and lung metastasis model

Male BALB/c nude mice aged 4–6 weeks old were purchased from the Medical Experimental Animal Center of Guangdong Province (Guangzhou, China). For the xenograft tumor growth model, 1 × 10^6^ SUNE-1 cells stably overexpressing miR-34c or negative control empty lenti-vector were suspended in 200 *μ*l PBS, and then subcutaneously injected into the dorsal flank of the nude mice. Tumor size was measured every 3 days, and tumor volumes were calculated. Four weeks later, the mice were killed, and the tumors were dissected and weighted. For the metastasis assay, SUNE-1 cells stably overexpressing miR-34c or negative control empty lenti-vector were suspended in PBS, and 1 × 10^6^ cells (200 *μ*l) were injected via the tail vein. Eight weeks later, the mice were killed, the lung tissues were fixed, paraffin embedded and 5 *μ*m tissue sections were stained with hematoxylin and eosin (H&E). The number of macroscopic and microscopic metastatic nodules in the lungs was counted. All animal research protocols were approved by the Institutional Animal Care and Use Ethics Committee.

### Luciferase reporter assay

The MET Wt and Mt 3′-UTR were generated and cloned into the *Xho*I and *Not*I restriction sites of the psiCHECK-2 luciferase reporter plasmid (Promega). For the luciferase assay, CNE-2 or SUNE-1 cells were seeded into 6-well plates the day before transfection, and then co-transfected with the MET Wt or Mt 3′-UTR reporter plasmids (2 *μ*g), and miR-34c mimic (50 nM) or miR-Ctrl (50 nM) using Lipofectamine 2000 reagent (Invitrogen). Renilla and firefly luciferase activities were measured using the Dual-Luciferase Reporter Assay System (Promega).

### Western blotting

Cells were lysed using RIPA buffer containing protease inhibitor cocktail (Fdbio Science, Hangzhou, China), and the protein concentrations were evaluated using the Pierce BCA Protein Assay Kit (Thermo Fisher Scientific, Waltham, MA, USA). Total proteins were separated on 10% SDS-PAGE gels, transferred to polyvinylidene fluoride membranes (Merck Millipore, Billerica, MA, USA) and the membranes were incubated with rabbit monoclonal anti-MET antibody (1 : 1000; Cell Signaling Technology, Beverly, MA, USA), and then incubated with anti-rabbit IgG secondary antibody (1 : 5000; Epitomics, Burlingame, CA, USA). An anti-*α*-tubulin antibody (1 : 1000; Sigma-Aldrich) was used as the loading control and the bands were detected by enhanced chemiluminescence.

### Immunofluorescent staining

Transfected CNE-2 or SUNE-1 cells were seeded onto coverslips (Thermo Fisher Scientific), and 24 h later, the coverslips were fixed, permeabilized and incubated with rabbit monoclonal anti-MET antibody (1 : 3000; Cell Signaling Technology), and then incubated with the Alexa Fluor 488 goat anti-rabbit IgG secondary antibody (Life Technologies, Carlsbad, CA, USA). Later, the cells were counterstained with 4′, 6-diamidino-2-phenylindole (DAPI) and images were captured using a confocal laser scanning microscope (Olympus FV1000, Olympus, Tokyo, Japan).

### DNA extraction and bisulfite pyrosequencing methylation analysis

Cells were treated with or without 2 *μ*M DAC (Sigma-Aldrich) for 72 h, replacing the drug and medium every 24 h. Genomic DNA was isolated from the cells using the EZ1 DNA Tissue Kit (Qiagen, Hilden, Germany), and 1 *μ*g of genomic DNA was subjected to bisulfite modification using the EpiTect Bisulfite Kit (Qiagen). Bisulfite pyrosequencing was performed with primers designed using PyroMark Assay Design Software 2.0 (Qiagen). The primer sequences used were as follows: PCR primers, 5′-GGGGTTTTAAGGAYGGTTGG-3′ (F) and 5′-ACCCCAAACCCTAAAACTAACTCT-3′ (R); sequencing primer, 5′-GGTAGTTTTAGTTATAGTTATT-3′. The sequencing reaction and quantitation of methylation were carried out using a PyroMark Q96 system (Qiagen). Percentage methylation was calculated by averaging across all CpG sites investigated.

### Statistical analysis

Data are presented as mean±S.D. All statistical analysis was performed using SPSS 16.0 software (IBM, Armonk, NY, USA). Two-tailed Student's *t*-tests were used for comparisons between groups, and *P*-values <0.05 were considered statistically significant.

## Abbreviations

NPC: nasopharyngeal carcinoma
MET: MET proto-oncogene
DAC: 5-aza-2′-deoxycytidine
miRNA: microRNA
3′-UTR: 3′-untranslated region

## References

[bib1] Jemal A, Bray F, Center MM, Ferlay J, Ward E, Forman D. Global cancer statistics. CA Cancer J Clin 2011; 61: 69–90. 21296855 10.3322/caac.20107

[bib2] McDermott AL, Dutt SN, Watkinson JC. The aetiology of nasopharyngeal carcinoma. Clin Otolaryngol Allied Sci 2001; 26: 82–92. 11309046 10.1046/j.1365-2273.2001.00449.x

[bib3] Lai SZ, Li WF, Chen L, Luo W, Chen YY, Liu LZ, et al. How does intensity-modulated radiotherapy versus conventional two-dimensional radiotherapy influence the treatment results in nasopharyngeal carcinoma patients? Int J Radiat Oncol Biol Phys 2011; 80: 661–668. 20643517 10.1016/j.ijrobp.2010.03.024

[bib4] Lee R, Feinbau R, Ambros V. A short history of a short RNA. Cell 2004; 116: S89–S92. 15055592 10.1016/s0092-8674(04)00035-2

[bib5] Bartel DP, Chen CZ. Micromanagers of gene expression: the potentially widespread influence of metazoan microRNAs. Nat Rev Genet 2004; 5: 396–400. 15143321 10.1038/nrg1328

[bib6] He L, Hannon GJ. MicroRNAs: small RNAs with a big role in gene regulation. Nat Rev Genet 2004; 5: 522–531. 15211354 10.1038/nrg1379

[bib7] Zamore PD, Haley B. Ribo-gnome: the big world of small RNAs. Science 2005; 309: 1519–1524. 16141061 10.1126/science.1111444

[bib8] Ambros V. The functions of animal microRNAs. Nature 2004; 431: 350–355. 15372042 10.1038/nature02871

[bib9] Esteller M. Non-coding RNAs in human disease. Nat Rev Genet 2011; 12: 861–874. 22094949 10.1038/nrg3074

[bib10] Brennecke J, Hipfner DR, Stark A, Russell RB, Cohen SM. Bantam encodes a developmentally regulated microRNA that controls cell proliferation and regulates the proapoptotic gene hid in Drosophila. Cell 2003; 113: 25–36. 12679032 10.1016/s0092-8674(03)00231-9

[bib11] Bartel DP. MicroRNAs: genomics, biogenesis, mechanism, and function. Cell 2004; 116: 281–297. 14744438 10.1016/s0092-8674(04)00045-5

[bib12] Calin GA, Croce CM. MicroRNA signatures in human cancers. Nat Rev Cancer 2006; 6: 857–866. 17060945 10.1038/nrc1997

[bib13] Volinia S, Calin GA, Liu CG, Ambs S, Cimmino A, Petrocca F, et al. A microRNA expression signature of human solid tumors defines cancer gene targets. Proc Natl Acad Sci USA 2006; 103: 2257–2261. 16461460 10.1073/pnas.0510565103 PMC1413718

[bib14] Lu J, Getz G, Miska EA, Alvarez-Saavedra E, Lamb J, Peck D, et al. MicroRNA expression profiles classify human cancers. Nature 2005; 435: 834–838. 15944708 10.1038/nature03702

[bib15] He L, Thomson JM, Hemann MT, Hernando-Monge E, Mu D, Goodson S, et al. A microRNA polycistron as a potential human oncogene. Nature 2005; 435: 828–833. 15944707 10.1038/nature03552 PMC4599349

[bib16] Esquela-Kerscher A, Slack FJ. Oncomirs – microRNAs with a role in cancer. Nat Rev Cancer 2006; 6: 259–269. 16557279 10.1038/nrc1840

[bib17] Calin GA, Croce CM. MicroRNA–cancer connection: the beginning of a new tale. Cancer Res 2006; 66: 7390–7394. 16885332 10.1158/0008-5472.CAN-06-0800

[bib18] Liu N, Jiang N, Guo R, Jiang W, He QM, Xu YF, et al. MiR-451 inhibits cell growth and invasion by targeting MIF and is associated with survival in nasopharyngeal carcinoma. Mol Cancer 2013; 12: 123. 24138931 10.1186/1476-4598-12-123 PMC3853142

[bib19] Liu N, Tang LL, Sun Y, Cui RX, Wang HY, Huang BJ, et al. MiR-29c suppresses invasion and metastasis by targeting TIAM1 in nasopharyngeal carcinoma. Cancer Lett 2013; 329: 181–188. 23142282 10.1016/j.canlet.2012.10.032

[bib20] Lu J, He ML, Wang L, Chen Y, Liu X, Dong Q, et al. MiR-26a inhibits cell growth and tumorigenesis of nasopharyngeal carcinoma through repression of EZH2. Cancer Res 2011; 71: 225–233. 21199804 10.1158/0008-5472.CAN-10-1850

[bib21] Lu J, Luo H, Liu X, Peng Y, Zhang B, Wang L, et al. MiR-9 targets CXCR4 and functions as a potential tumor suppressor in nasopharyngeal carcinoma. Carcinogenesis 2014; 35: 554–563. 24170200 10.1093/carcin/bgt354

[bib22] Liu N, Chen NY, Cui RX, Li WF, Li Y, Wei RR, et al. Prognostic value of a microRNA signature in nasopharyngeal carcinoma: a microRNA expression analysis. Lancet Oncol 2012; 13: 633–641. 22560814 10.1016/S1470-2045(12)70102-X

[bib23] Chen HC, Chen GH, Chen YH, Liao WL, Liu CY, Chang KP, et al. MicroRNA deregulation and pathway alterations in nasopharyngeal carcinoma. Br J Cancer 2009; 100: 1002–1011. 19293812 10.1038/sj.bjc.6604948 PMC2661776

[bib24] Li T, Chen JX, Fu XP, Yang S, Zhang Z, Chen K, et al. MicroRNA expression profiling of nasopharyngeal carcinoma. Oncol Rep 2011; 25: 1353–1363. 21373758 10.3892/or.2011.1204

[bib25] Luo Z, Zhang L, Li Z, Li X, Li G, Yu H, et al. An *in silico* analysis of dynamic changes in microRNA expression profiles in stepwise development of nasopharyngeal carcinoma. BMC Med Genom 2012; 5: 3. 10.1186/1755-8794-5-3 PMC3293045 22260379

[bib26] Dong F, Lou D. MicroRNA-34b/c suppresses uveal melanoma cell proliferation and migration through multiple targets. Mol Vis 2012; 18: 537–546. 22419847 PMC3298424

[bib27] Nadal E, Chen G, Gallegos M, Lin L, Ferrer-Torres D, Truini A, et al. Epigenetic inactivation of microRNA-34b/c predicts poor disease-free survival in early-stage lung adenocarcinoma. Clin Cancer Res 2013; 19: 6842–6852. 24130071 10.1158/1078-0432.CCR-13-0736 PMC4161219

[bib28] Hagman Z, Haflidadottir BS, Ansari M, Persson M, Bjartell A, Edsjo A, et al. The tumour suppressor miR-34c targets MET in prostate cancer cells. Br J Cancer 2013; 109: 1271–1278. 23922103 10.1038/bjc.2013.449 PMC3778300

[bib29] Yang S, Li Y, Gao J, Zhang T, Li S, Luo A, et al. MicroRNA-34 suppresses breast cancer invasion and metastasis by directly targeting Fra-1. Oncogene 2013; 32: 4294–4303. 23001043 10.1038/onc.2012.432

[bib30] Toyota M, Suzuki H, Sasaki Y, Maruyama R, Imai K, Shinomura Y, et al. Epigenetic silencing of microRNA-34b/c and B-cell translocation gene 4 is associated with CpG island methylation in colorectal cancer. Cancer Res 2008; 68: 4123–4132. 18519671 10.1158/0008-5472.CAN-08-0325

[bib31] Hagman Z, Larne O, Edsjo A, Bjartell A, Ehrnstrom RA, Ulmert D, et al. MiR-34c is downregulated in prostate cancer and exerts tumor suppressive functions. Int J Cancer 2010; 127: 2768–2776. 21351256 10.1002/ijc.25269

[bib32] Kumamoto K, Spillare EA, Fujita K, Horikawa I, Yamashita T, Appella E, et al. Nutlin-3a activates p53 to both down-regulate inhibitor of growth 2 and up-regulate mir-34a, mir-34b, and mir-34c expression, and induce senescence. Cancer Res 2008; 68: 3193–3203. 18451145 10.1158/0008-5472.CAN-07-2780 PMC2440635

[bib33] Yu F, Jiao Y, Zhu Y, Wang Y, Zhu J, Cui X, et al. MicroRNA 34c gene down-regulation via DNA methylation promotes self-renewal and epithelial-mesenchymal transition in breast tumor-initiating cells. J Biol Chem 2012; 287: 465–473. 22074923 10.1074/jbc.M111.280768 PMC3249099

[bib34] Kress TR, Cannell IG, Brenkman AB, Samans B, Gaestel M, Roepman P, et al. The MK5/PRAK kinase and Myc form a negative feedback loop that is disrupted during colorectal tumorigenesis. Mol Cell 2011; 41: 445–457. 21329882 10.1016/j.molcel.2011.01.023

[bib35] Birchmeier C, Birchmeier W, Gherardi E, Vande WG. Met, metastasis, motility and more. Nat Rev Mol Cell Biol 2003; 4: 915–925. 14685170 10.1038/nrm1261

[bib36] Trusolino L, Bertotti A, Comoglio PM. MET signalling: principles and functions in development, organ regeneration and cancer. Nat Rev Mol Cell Biol 2010; 11: 834–848. 21102609 10.1038/nrm3012

[bib37] Gastaldi S, Sassi F, Accornero P, Torti D, Galimi F, Migliardi G, et al. Met signaling regulates growth, repopulating potential and basal cell-fate commitment of mammary luminal progenitors: implications for basal-like breast cancer. Oncogene 2013; 32: 1428–1440. 22562252 10.1038/onc.2012.154

[bib38] Qian CN, Guo X, Cao B, Kort E, Lee CC, Chen J, et al. Met protein expression level correlates with survival in patients with late-stage nasopharyngeal carcinoma. Cancer Res 2002; 62: 589–596. 11809714

[bib39] Li Y, Zhang S, Tang Z, Chen J, Kong W. Silencing of c-Met by RNA interference inhibits the survival, proliferation, and invasion of nasopharyngeal carcinoma cells. Tumour Biol 2011; 32: 1217–1224. 21922276 10.1007/s13277-011-0225-y

[bib40] Croce CM. Causes and consequences of microRNA dysregulation in cancer. Nat Rev Genet 2009; 10: 704–714. 19763153 10.1038/nrg2634 PMC3467096

[bib41] Esteller M. Cancer epigenomics: DNA methylomes and histone-modification maps. Nat Rev Genet 2007; 8: 286–298. 17339880 10.1038/nrg2005

[bib42] Korbler T, Grskovic M, Dominis M, Antica M. A simple method for RNA isolation from formalin-fixed and paraffin-embedded lymphatic tissues. Exp Mol Pathol 2003; 74: 336–340. 12782023 10.1016/s0014-4800(03)00024-8

[bib43] Livak KJ, Schmittgen TD. Analysis of relative gene expression data using real-time quantitative PCR and the 2(−Delta Delta C (T)) method. Methods 2001; 25: 402–408. 11846609 10.1006/meth.2001.1262

[bib44] Kingston RE, Chen CA, Okayama H. Calcium phosphate transfection. Curr Protoc Immunol 2001; Chapter 10 Unit 10.13 doi:10.1002/0471142735.im1013s31. 10.1002/0471142735.im1013s31 18432676

